# Revealing the Connection Between Hemodialysis and Sexual Physiology in Women With End-Stage Renal Disease

**DOI:** 10.7759/cureus.35184

**Published:** 2023-02-19

**Authors:** Nargis Abdul Rahman, Mansoor Ghani, Samina Kausar, Ayesha Sadiqa, Asma Khalid

**Affiliations:** 1 Nursing, Institute of Nursing, University of Health Sciences, Lahore, PAK; 2 Internal Medicine, University of Health Sciences, Lahore, PAK; 3 Physiology, CMH (Combined Military Hospitals) Lahore Medical College and Institute of Dentistry, Lahore, PAK; 4 Nursing, Gulfreen Nursing College, Avicenna Medical College Lahore, Lahore, PAK

**Keywords:** quality of life (qol), fsfi score, pre-dialysis ckd, women sexual health, chronic kidney disease (ckd), sexual physiology., end-stage renal disease, hemodialysis, female sexual health, female sexuality

## Abstract

Introduction: In the recent past, the procedure of hemodialysis has frequently been opted for patients with end-stage renal disease (ESRD) around the globe. In such patients, the concern of sexual dysfunction is highly prevalent, which causes psychological as well as social deterioration in these patients. Wretchedly, this issue has been ignored in developing countries like Pakistan because of social and cultural constraints.

Objectives: The aim was to measure and compare Female Sexual Functions of Dialysis (FSFI) scores among three comparative groups: healthy controls, pre-dialysis patients, and hemodialysis patients.

Methods: A comparative cross-sectional study was carried out with 60 females aged 22-50 years in which 20 were healthy (controls) and 40 were patients with ESRD; of these 40, 20 were taking only oral medicines (pre-dialysis) and 20 were also receiving hemodialysis (hemodialysis). Married women who could read Urdu and were living with live spouses were included, and those with any psychological or psychiatric illness were excluded. Data was collected through a Likert-scaled questionnaire, Urdu translation of the FSFI questionnaire, and scores of each domain were analyzed. Single-tail one-way ANOVA was used to observe the significant difference among the three comparative groups.

Results: A strong statistical difference was observed among the hemodialysis, pre-dialysis, and healthy control groups when these three study groups were compared for the mean scores of all related domains of FSFI questtionarie. In each female sexual domain, i.e. Desire, Arousal, Lubrication, Orgasm, Satisfaction, and Pain, the diseased groups (pre-dialysis and hemodialysis) showed lower sexual scores than the healthy group. The lowest scores were observed in the pre-dialysis group (16.4 ± 6.8) and the highest were noticed in the healthy group (29.9 ± 1.8); the hemodialysis group (23.3 ± 5.0) expressed a moderate pattern of scores in each sexual domain.

Conclusion: ESRD female patients who were receiving hemodialysis along with routine oral medications showed improved sexual physiology (with better FSFI scores) compared to those who were without hemodialysis.

## Introduction

Chronic kidney disease (CKD) is a global medical concern as it not only declines the quality of life but acts as a burden on the healthcare system because of its expenses [1]. More than that, it is one of the major causes of premature mortality around the globe [2]. Perico and Remuzzi reported in 2017 that about 10% of the world’s population is affected with CKD and millions die each year because of the unaffordability of its medical treatment [3]. If CKD is ignored, it can turn into a more serious condition called end-stage renal disease (ESRD), which can only be treated through costly procedures like hemodialysis or kidney transplant [4]. Studies report that ESRD derails many human physiological systems [5-6]. Literature has proven the association of sexual dysfunctionality with CKD, as well as ESRD, and this vital aspect of physiology mainly remained neglected compared to other physical health issues. [7]

A higher incidence rate of sexual dysfunctionality has been reported in female hemodialysis patients than in their counterpart males. According to Karabulutlu et al., approximately 40% of males and 55% of females who have been receiving hemodialysis experience trouble in getting orgasms, even though more paucity has been found in the literature related to female sexual dysfunction in patients of ESRD [8]. Sexual dysfunction affects the life of women negatively [9]. Approximately 84% of American women with ESRD have been found to suffer from sexual dysfunction [10].

Basok et al. declared that undoubtedly losing sexuality may reduce self-confidence in a person and this can lead to inconvenience in marital and family relationships [11]. The psychological and social impact of sexual dysfunction on a patient’s well-being cannot be undermined [12], but this problem has been more ignored in societies like Pakistan where social and cultural setup poses many challenges.

Bayram et al. documented that sexual dysfunction in females can be assessed through various physiological traits such as difficulty in sexual desire, arousal, vaginal lubrication, orgasm achievement, and pain during intercourse [13]. The aim of the current study is to measure and compare the Female Sexual Functions of Dialysis (FSFI) scores among three comparative study groups, i.e. healthy controls, pre-dialysis patients, and hemodialysis patients.

## Materials and methods

A comparative cross-sectional study was carried out at the Institute of Nursing, University of Health Sciences (UHS), Lahore, Pakistan. A total of 60 women aged 22-50 years participated in the study. A non-probability convenient sampling technique was employed to select the study population after obtaining ethical approval from the Ethical Review Committee of the University of Health Sciences Lahore (Approval number: UHS/REG-18/ERC/3778). The duration of the study was six months from November 2016 to April 2017.

The study population consisted of two major categories: 20 were healthy (labeled as the Control Group) and 40 were patients with ESRD. All the selected patients were under treatment for more than three months in the nephrology department of four tertiary care hospitals in Lahore, Pakistan, of which two were from the private sector, i.e. Sheikh Zayed Hospital and Shalamar Hospital, and two were public sector hospitals, i.e. Mayo Hospital and Lahore General Hospital.

The patients were further subdivided into two groups based on the difference in their therapy, 20 were those who were using only oral medicines for treatment (labeled as the Pre-dialysis Group), and the other 20 were also receiving hemodialysis for their treatment (labeled as the Hemodialysis Group). All the patients had only diabetes mellitus type II and hypertension as co-morbidities. Women who were married and living with alive spouses, and could read and understand Urdu were included in the study, while those who were more than 50 years of age and with any psychological or psychiatric illness were excluded from the study.

Informed consent was taken from each of the participants of the study. The results were obtained by using a pre-validated Urdu version of the FSFI questionnaire after seeking permission from the authors who actually translated this FSFI questionnaire into Urdu, through email (please find both the Urdu questionnaire as well as its English translation in the Annexure). The original English FSFI questionnaire was given as supplementary material in a previous randomized control study by Reed et al. published in 2014, in which the sexual functionality of midlife women was evaluated [14].

The obtained scores of the individual items of the FSFI study tool were analyzed in IBM SPSS Statistics for Windows, Version 21.0 (Released 2012; IBM Corp., Armonk, New York, United States) for descriptive investigations. One-way ANOVA was used as a statistical test to observe the significant difference among the three comparative study groups and a 95% confidence interval was considered statistically significant.

## Results

All 20 hemodialysis patients were receiving hemodialysis procedures with an average frequency of 2.45 ± 0.5 dialyses in one week. The skewness of the age was towards the younger population of the study participants. The Hemodialysis Group was more aged (37.85 ± 6.58) compared to the Pre-dialysis Group (36.8 ± 10.4) and Control Group (35.65 ± 6.98) groups; however, the range of age was wider in the Pre-dialysis Group (22 - 50) compared to the Hemodialysis Group (28 - 47) and the Control Group (25 - 49). The mean length of marriage duration (years) was maximum in the Hemodialysis Group (16.85 ± 8.9) and least in the Control Group (10.04 ± 6.82). Moreover, the Pre-dialysis Group showed the widest range of marriage duration in months (0.83-30). The Hemodialysis Group expressed a 56.25% wider range of duration after diagnosis with ESRD compared to the Pre-dialysis Group; also, the exhibited mean duration after their diagnosis with ESRD was 56.17% more in the Hemodialysis Group than the Pre-dialysis Group (Table 1).

**Table 1:** Baseline statistics of all the participants in each study group DM: diabetes mellitus type II; HTN: hypertension; NA: not applicable

Study Groups	Number (n)	Age (years)		Duration of marriage (years)		Co-morbidity n (%)	Duration after diagnosis (month)	
		Mean ± SD	Range (min. – max.)	Mean ± SD	Range (min. – max.)		Mean ± SD	Range (min. – max.)
Hemodialysis Group	20	37.85 ± 6.58	28 - 47	16.85 ± 8.9	4.0 - 30	7 (35)	26.55 ± 18.79	4.0 - 54
						5 (25) with DM 2 (10) with HTN		
Pre-dialysis Group	20	36.8 ± 10.4	22 - 50	14.84 ± 10.3	0.833 - 30	7 (35)	17 ± 9.9	4.0 - 36
						5 (25) with DM 2 (10) with HTN		
Healthy Control Group	20	35.65 ± 6.98	25 - 49	10.04 ± 6.82	0.75 - 24	nil	NA	
Total study population	60	36.77 ± 8.10	22 - 50	13.92 ± 9.24	0.75 - 30	14 (23)	NA	

Compared to the Control Group, the Pre-dialysis Group and the Hemodialysis Group expressed 42.5% and 21.25% less sexual desire, respectively. Similarly, when the participants were asked about their level of sexual desire, the Pre-dialysis Group revealed a 43.83% reduced response compared to the Control Group, while the Hemodialysis Group displayed only 13.69% less response compared to the Control Group. The Pre-dialysis Group responded 46.34% less arousal frequency during the sexual act compared to the Control Group, and for the same question, the Hemodialysis Group expressed only 9.75% less response than the Control Group. Likewise, when they were asked about their rate of arousal during intercourse, the Pre-dialysis Group declared a 43.73% reduced response compared to the Control Group and the Hemodialysis Group showed only a 20% decreased response than the Control Group. When the participants were questioned regarding their self-confidence in becoming aroused during intercourse, the Pre-dialysis Group expressed a 53.48% lesser response than the Control Group and the Hemodialysis Group showed only a 20.90% decline compared to the Control Group. Along the same lines, regarding the satisfaction level of arousal in intercourse, the Pre-dialysis Group responded about 43.67% less in comparison to the Control Group and the Hemodialysis Group showed 22.98% less than the Control Group (Table 2).

**Table 2:** Comparison of study participants’ responses over the four weeks of the study to each item of the FSFI questionnaire FSFI: Female Sexuality Function Index

Domains of FSFI	Item of FSFI related to each domain	Response of each study group to the FSFI questionnaire in Mean ± SD		
		Hemodialysis Group	Pre-dialysis Group	Control Group
Desire	1: How often did you feel sexual desire?	3.15 ± 0.99	2.3 ± 0.93	4.00 ± 0.46
	2: How would you rate your level of sexual desire?	3.15 ± 0.81	2.05 ± 0.83	3.65 ± 0.67
Arousal	3: How often did you feel sexually aroused during sexual activity?	3.70 ± 0.98	2.20 ± 1.15	4.10 ± 0.64
	4: Rate your level of sexual arousal during intercourse?	3.00 ± 0.79	2.11 ± 1.20	3.75 ± 0.85
	5: How confident were you about becoming sexually aroused during intercourse?	3.40 ± 1.05	2.00 ± 1.11	4.30 ± 0.92
	6: How often have you been satisfied with your arousal during intercourse?	3.35 ± 0.88	2.45 ± 1.39	4.35 ± 0.67
Lubrication	7: How often did you become lubricated during intercourse?	3.21 ± 0.71	2.15 ± 1.18	4.20 ± 0.41
	8: How difficult was it to become lubricated during intercourse?	2.85 ± 1.14	1.65 ± 1.23	4.55 ± 0.69
	9: How often did you maintain your lubrication until completion of intercourse?	3.10 ± 1.07	1.83 ± 1.42	3.90 ± 0.72
	10: How difficult was it to maintain your lubrication until completion of intercourse?	2.95 ± 1.15	2.05 ± 1.43	4.50 ± 0.95
Orgasm	11: When you had intercourse, how often did you reach orgasm?	3.20 ± 1.01	2.10 ± 1.37	4.45 ± 0.51
	12: When you had intercourse, how difficult was it for you to reach orgasm?	2.60 ± 1.19	1.95 ± 1.28	4.35 ± 0.93
	13: How satisfied were you with your ability to reach orgasm during intercourse?	3.15 ± 0.99	2.35 ± 1.35	4.45 ± 0.76
Satisfaction	14: How satisfied have you been with the amount of emotional closeness during sexual activity between you and your partner?	3.05 ± 1.05	2.05 ± 1.05	4.30 ± 0.66
	15: How satisfied have you been with your sexual relationship with your partner?	3.15 ± 0.88	2.10 ± 0.79	4.10 ± 0.45
	16: How satisfied have you been with your overall sexual life?	3.20 ± 0.52	2.10 ± 0.79	4.05 ± 0.60
Pain	17: How often did you experience pain during vaginal penetration?	3.45 ± 1.57	2.80 ± 1.79	3.55 ± 0.89
	18: How often did you experience pain following vaginal penetration?	4.00 ± 1.34	3.45 ± 1.67	4.65 ± 0.59
	19: How would you rate your level of pain during/following vaginal penetration?	3.90 ± 0.97	3.10 ± 1.52	4.15 ± 0.37

Taking about the frequency of becoming lubricated during intercourse, the Pre-dialysis Group responded 48.8% lesser than the Control Group and the Hemodialysis Group answered only 23.57% lesser compared to the Control Group. When the study participants were asked about the difficulty level of becoming lubricated in intercourse, the Pre-dialysis Group was 63.73% lower than the Control Group, while the Hemodialysis Group was only 37.36% lower compared to the Control Group. When they were questioned about the frequency of maintaining their lubrication in intercourse, the Pre-dialysis Group was 53.07% lower in their responses compared to the Control Group, while the Hemodialysis Group was only 20.51% lower than the Control Group. Similarly, related to the difficulty level of maintenance of lubrication during the entire intercourse, again the Pre-dialysis Group was 54.44% lower than the Control Group whereas the Hemodialysis Group was only 28.08% lower than the Control Group. Response to the orgasm-related question (frequency of acquiring orgasm in intercourse), the Pre-dialysis Group expressed 52.81% lesser than the Control Group and the Hemodialysis Group replied only 28.08% lower than the Control Group. Likewise, regarding the difficulty level to reach orgasm, the Pre-dialysis Group declared 55.17% lesser than the Control Group and the Hemodialysis Group responded only 40.23% lower than the Control Group. Concerning the satisfaction level of orgasm, the Pre-dialysis Group responded 47.19% lesser compared to the Control Group while the Hemodialysis Group answered only 29.21% lesser than the Control Group (Table 2).

When participants were inquired about the satisfaction level of emotional closeness with their partner in intercourse, the Pre-dialysis Group responded 52.32% lower than the Control Group and the Hemodialysis Group reacted only 29.06% lesser than the Control Group. Similarly, when the study groups were asked about the satisfaction level of their sexual relationship with their partners, the Pre-dialysis Group replied 48.78% lower than the Control Group and the Hemodialysis Group replied only 23.17% lesser than the Control Group. In the same connection, when they were questioned about the overall satisfaction level of their sexual life, the Pre-dialysis Group responded 48.14% lesser than the Control Group while the Hemodialysis Group responded only 20.98% lesser than the Control Group. Regarding pain experienced during vaginal penetration, the Pre-dialysis Group experienced 21.13% lesser pain compared to the Control Group whereas the Hemodialysis Group felt only 2.82% reduced pain compared to the Control Group. Responses related to pain after the vaginal penetration revealed that the Pre-dialysis Group reported 25.8% lesser pain than the Control Group while the Hemodialysis Group showed only 13.97% reduced pain than the Control Group. When all the participants were asked to rate their pain level during or after the vaginal penetration, the Pre-dialysis Group rated 25.3% lesser pain than the Control Group, and the Hemodialysis Group rated 22.64% decreased pain than the Control Group (Table 2).

A strong statistical difference (p = 0.000) was found when the mean scores of three study groups were compared to assess the ‘Desire’ domain of FSFI. Here the Hemodialysis Group expressed 17.4% lesser sexual desire than the Control Group, while the Pre-dialysis Group showed 43.5% lesser sexual desire compared to the Control Group. The comparison among the groups for their mean ‘sexual arousal’ scores revealed a strong statistically significant difference (p = 0.000), where the Hemodialysis Group showed 20% lesser sexual arousal compared to the Control Group, whereas the Pre-dialysis Group exhibited 40% lesser sexual arousal than the Control Group. Similarly, when the three groups were compared for their average scores of ‘lubrication in intercourse’, again a significant difference (p = 0.000) was observed. The Hemodialysis Group displayed 29.4% lesser sexual lubrication than the Control Group, while the Pre-dialysis Group displayed much lesser, i.e. 55%, decreased sexual lubrication compared to the Control Group (Table 3). 

**Table 3:** Statistical difference of average scores of responses of all study groups to each domain of the FSFI Questionnaire *P < 0.05, significant FSFI: Female Sexuality Function Index

Domain	Comparative Groups	Number (n)	Mean Score ± SD	Sore Range (min. - max.)	p-value
Desire	Hemodialysis	20	3.8 ± 0.9	1.8 - 4.8	0.000*
	Pre-dialysis	20	2.6 ± 1.0	1.2 - 4.2	
	Control	20	4.6 ± 0.5	3.6 - 5.4	
Arousal	Hemodialysis	20	4.0 ± 1.0	1.8 - 5.1	0.000*
	Pre-dialysis	20	2.6 ± 1.4	0 - 4.5	
	Control	20	5.0 ± 0.7	3.9 - 6.0	
Lubrication	Hemodialysis	20	3.6 ± 0.9	0.6 - 4.8	0.000*
	Pre-dialysis	20	2.3 ± 1.3	0 - 4.2	
	Control	20	5.1 ± 0.5	3.9 - 5.7	
Orgasm	Hemodialysis	20	3.6 ± 1.2	0 - 4.8	0.000*
	Pre-dialysis	20	2.6 ± 1.4	0 - 4.8	
	Control	20	5.3 ± 0.5	4.4 - 6.0	
Satisfaction	Hemodialysis	20	3.8 ± 0.9	1.6 - 5.6	0.000*
	Pre-dialysis	20	2.5 ± 1.0	0.8 - 4.4	
	Control	20	5.0 ± 0.4	4.4 - 6.0	
Pain	Hemodialysis	20	4.5 ± 1.3	0 - 5.6	0.013*
	Pre-dialysis	20	3.7 ± 1.7	0 - 5.6	
	Control	20	4.9 ± 0.4	4.4 - 5.6	
Total score	Hemodialysis	20	23.3 ± 5.0	7.6 - 29.9	0.000*
	Pre-dialysis	20	16.4 ± 6.8	2.0 - 23.6	
	Control	20	29.9 ± 1.8	26.2 - 33.2	

Likewise, when these three study groups were compared for their mean score in the domain of ‘orgasm’, a very significant statistical difference (p = 0.000) was noted. The Hemodialysis Group revealed a 32% lesser orgasm score compared to the Control Group and the Pre-dialysis Group expressed a 51% decreased orgasm score than the Control Group. An almost similar pattern was observed with the ‘sexual satisfaction’ domain among the three comparative groups. A high statistical difference (p = 0.000) was seen among the three groups for sexual satisfaction average scores. Here again, less difference was found between the Hemodialysis Group (24% lesser sexually satisfied) and the Control Group, and a greater difference was noted between the Pre-dialysis Group (50% lesser sexually satisfied) and the Control Group.

The statistical difference related to sexual pain among the three comparative study groups exhibited a significant difference with p = 0.013. In this comparison, the Hemodialysis Group expressed 8.16% less painful sex than the Control Group and the Pre-dialysis Group showed 25.5% reduced pain levels in their sexual intercourse. Thus overall, a strong statistical difference (p = 0.000) was observed among the Hemodialysis Group, the Pre-dialysis Group, and the Control Group for their mean sexual function scores. The Hemodialysis Group showed 22.1% less sexual functioning than the Control Group, while the Pre-dialysis Group explored a 45.2% reduced sexual function compared to the Control Group (Table 3).

## Discussion

A normal sexual relationship is considered one of the basic human needs. Literature reported a strong association of sexual physiology with quality of life [15]. Impaired sexual functions can lead to damage to the individual’s confidence, sense of wholeness, social relation, and marital aspect of women [16]. Studies also explored that sexual dysfunction is one of the offshoots among patients of CKD, especially those with ESRD [7,17]. A Turkish study by Bayram et al. reported reduced sexual function in female patients with renal disorder [13]. A study found that 46% of ESRD female patients showed a decrease in sexual desire [18]. In the same regard, a Spanish study conducted with female ESRD patients reported 67.9% sexual dysfunctionality in the study population [19], while according to the current study, the FSFI scores were 44.0%, 67.3%, and 82.5% for pre-dialysis, hemodialysis, and healthy groups respectively. Likewise, a Swedish study with a similar target population also reported that women in the pre-dialysis group expressed fewer sexual functions than women in the dialysis group [18].

A similar Turkish study by Basok et al. also verified that dialysis increases the sexual functional index (SFI) in women with CKD or ESRD though it did not touch the SFI of a healthy group. They also observed that improved sexual health in such women uplifted the quality of their life [11]. Another study in Wisconsin also affirmed a highly significant positive association between quality of life and sexual health in women [20].

In his study, Montgomery observed that the female patients of ESRD expressed better scores with respect to ‘frequency of sexual desire’ and ‘intensity of desire’ after receiving the dialysis treatment, even though it’s not the same as found in healthy controls [21]. Along the same lines, the present study showed that compared to the healthy group, the pre-dialysis group expressed 42.5% lesser sexual desire and the hemodialysis group showed 21.25% lesser sexual desire than the healthy group. It has been hypothesized that the reason behind improved sexual physiology could be the filtration of waste and by-products from the blood through dialysis, which ultimately led to sex hormonal correction as well as psychological well-being, which are crucial factors for sexual arousal [22]. Wallen and Lloyd announced a statistical association of sexual arousal with the duration and frequency of dialysis [23]. Soykan et al. also stated a significant association of sexual desire with the duration and frequency of dialysis in patients with CKD [24]. A similar study by Steele et al. exhibited that women with CKD showed an upsurge in sexual arousal by up to 54% after dialysis [25].

Azevedo et al. found that a pre-dialysis study group of female patients with CKD experienced less frequent vaginal lubrication during sexual intercourse [19], which is considered one of the prime domains of FSFI. For the same domain, the present study concluded that the pre-dialysis group expressed 48.8% lesser than the control group and the hemodialysis group expressed only 23.57% lesser than the control group.

Similarly, orgasm is another domain of FSFI, Strippoli observed that pre-dialyzed patients reported less frequent orgasm states during their intercourse [17]. The present study also found parallel results in the same connection, as the hemodialysis group revealed a 32% lower orgasm score compared to the controls while the pre-dialysis group expressed a 51% decreased orgasm score than controls. Thus the present study is consistent with the findings of Basok et al. [11], Oksuz and Malhan [16], and Steele [25] and that in all the domains of FSFI, the pre-dialysis group expressed the least scores.

A limitation of the study was that the participants belonged to only four hospitals in Lahore city of Punjab, Pakistan. A more diverse and large study population with an increased number of study participants from various cities and hospitals of the state could reflect more generalized and detailed findings.

## Conclusions

A strong statistical difference was observed among hemodialysis, pre-dialysis, and healthy groups when these three study groups were compared for the mean scores of all related domains of the FSFI. In all the domains of the FSI, the diseased groups (pre-dialysis and hemodialysis) showed lower sexual scores than the healthy group. The least sexual scores were observed in the pre-dialysis group and the highest scores were noticed in the healthy group; the hemodialysis group expressed a moderate pattern of scores in each sexual domain.

ESRD female patients who were receiving hemodialysis along with routine oral medications showed improved sexual physiology (with better scores on FSFI) compared to those who were without hemodialysis.

## Figures and Tables

**Figure 1:**
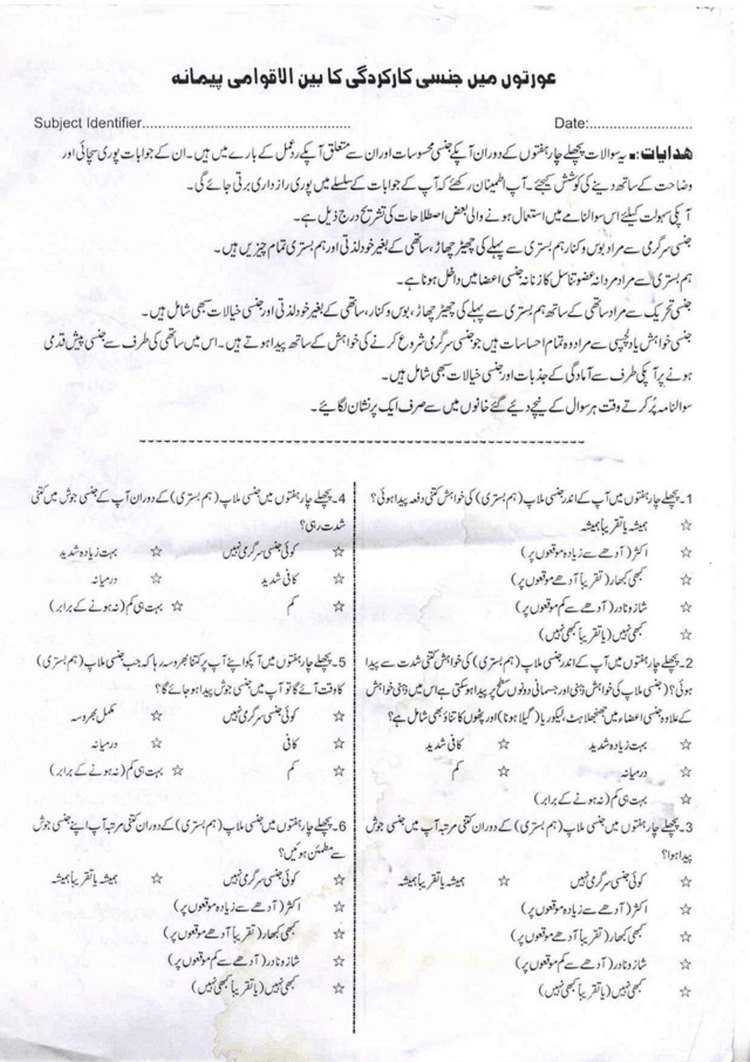
The Urdu FSFI questionnaire (page 1) FSFI: Female Sexuality Function Index Permission for use received from the author via email.

**Figure 2:**
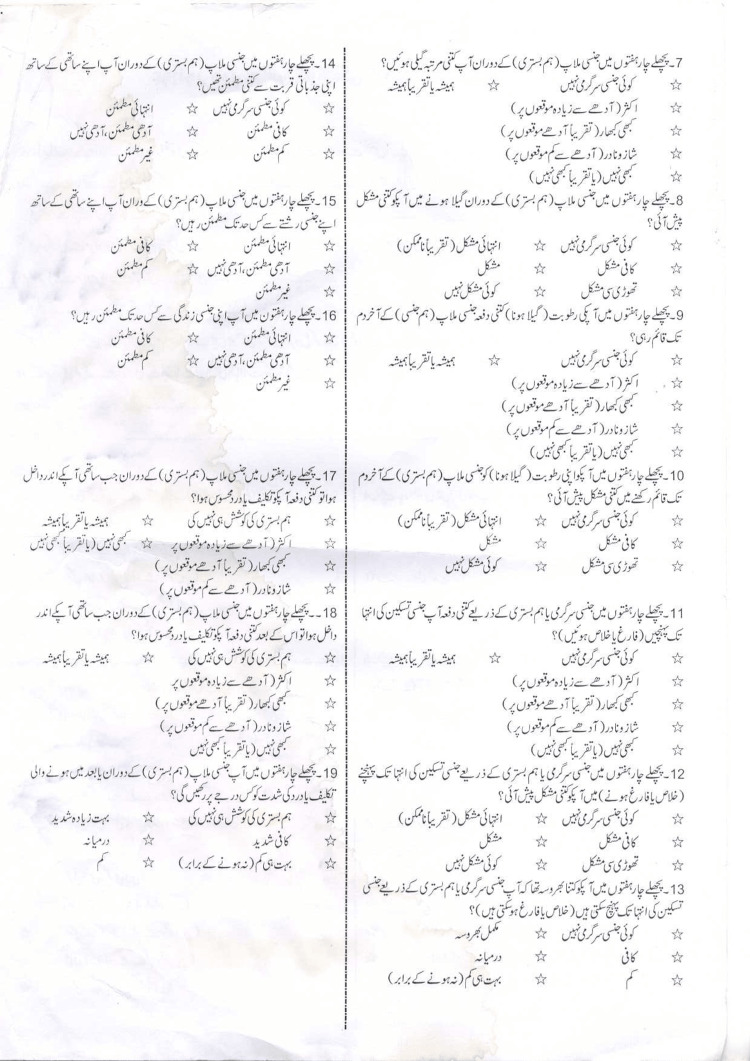
The Urdu FSFI questionnaire (page 2) FSFI: Female Sexuality Function Index Permission for use received from the author via email.

**Figure 3:**
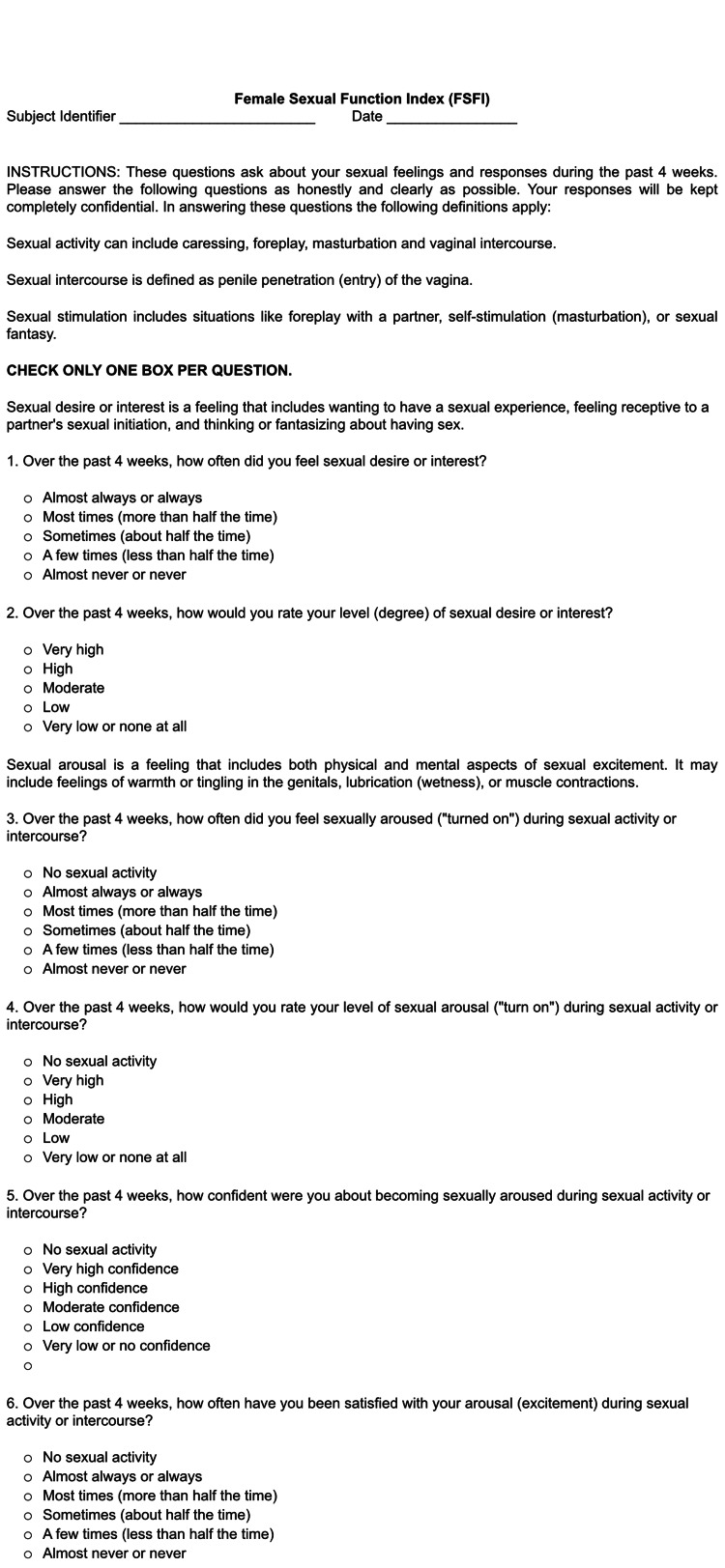
FSFI Urdu questionnaire translated into English (page 1) FSFI: Female Sexuality Function Index

**Figure 4:**
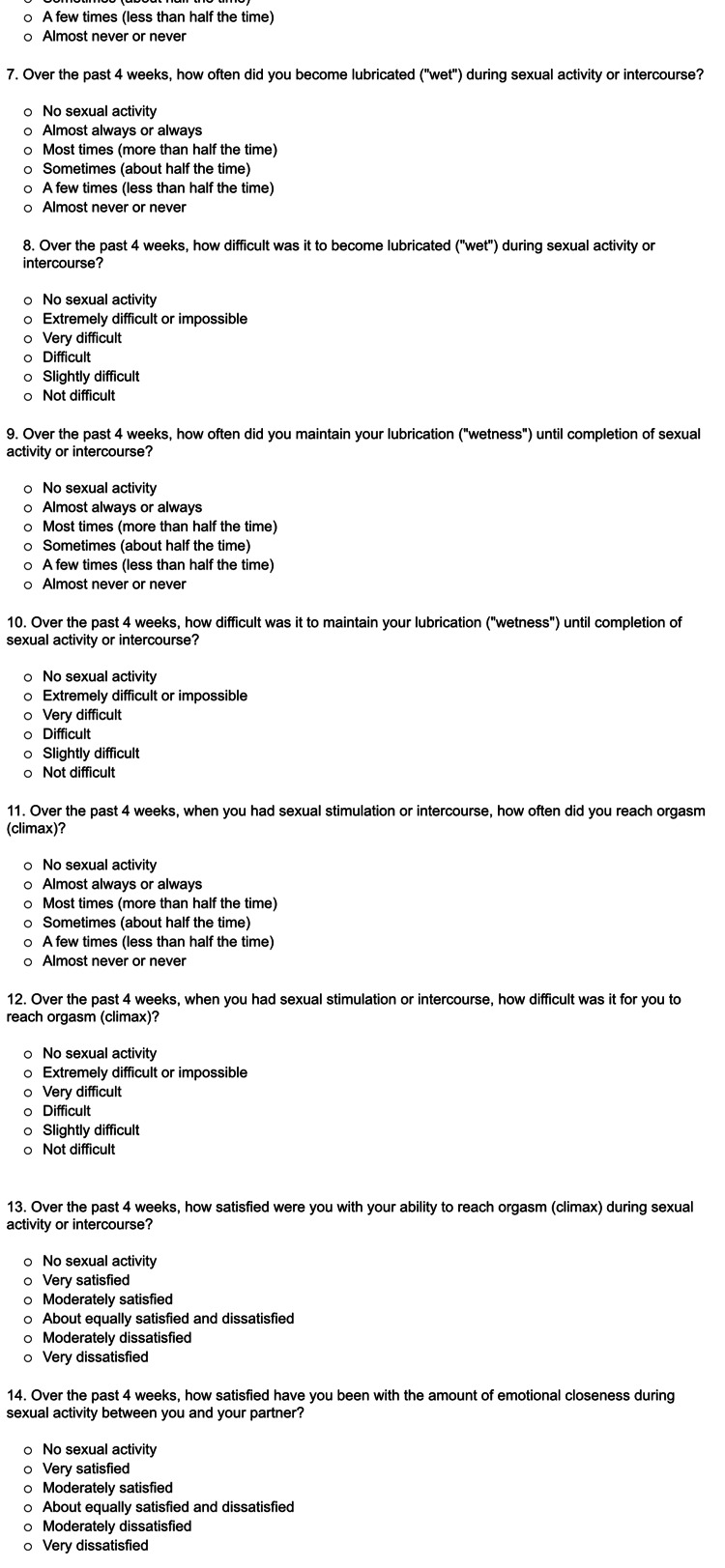
FSFI Urdu questionnaire translated into English (page 2) FSFI: Female Sexuality Function Index

**Figure 5:**
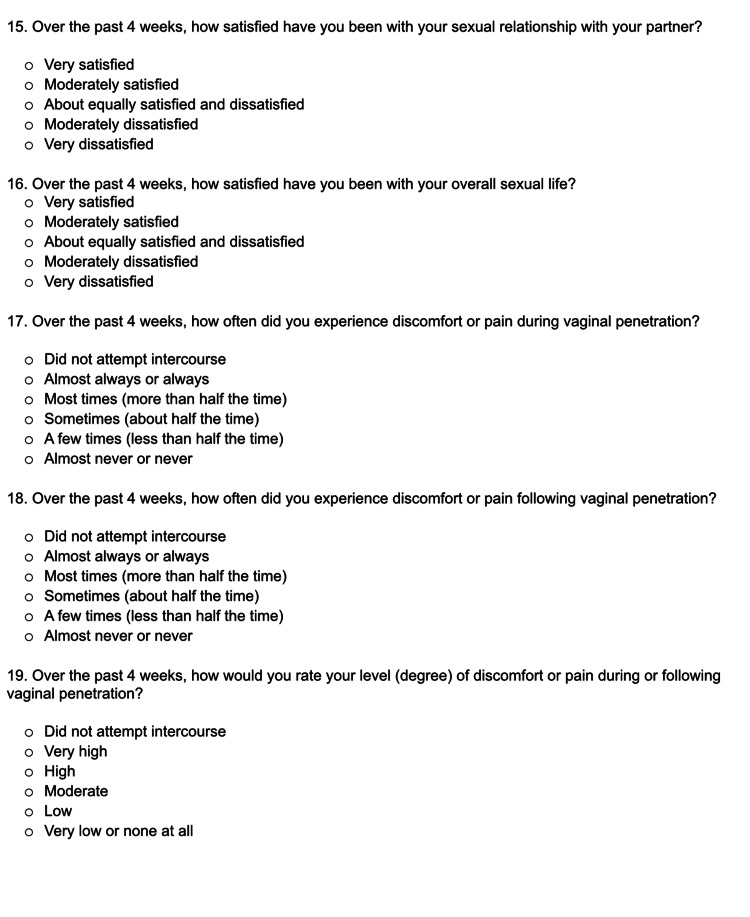
FSFI Urdu questionnaire translated into English (page 3) FSFI: Female Sexuality Function Index
